# Association of triglyceride-glucose index with ischemic stroke recurrence in nondiabetic patients with small vessel occlusion: a multicenter hospital-based prospective cohort study

**DOI:** 10.1186/s12933-022-01693-4

**Published:** 2022-11-17

**Authors:** Li Wu, Jinmao Zhu, Chenghao Li, Juehua Zhu, Zheng Dai, Yongjun Jiang

**Affiliations:** 1 Department of Neurology, The Second Affiliated Hospital of Guangzhou Medical University, 250 Changgang East Road, Guangzhou, 510260 China; 2 Department of Radiology, The Second Affiliated Hospital of Guangzhou Medical University, 250 Changgang East Road, Guangzhou, 510260 China; 3 Department of Neurology, The First Affiliated Hospital of Soochow University, 899 Pinghai Road, Suzhou, 215300 China; 4 Department of Neurology, Wuxi People’s Hospital, 299 Qingyang Road, Wuxi, 214023 China

**Keywords:** Triglyceride-glucose index, Insulin resistance, Stroke recurrence, Small vessel occlusion, Nondiabetic, Cerebral small vessel disease

## Abstract

**Background:**

Triglyceride-glucose (TyG) index is a simple and reliable surrogate marker of insulin resistance. Elevated TyG index was related to stroke recurrence. This study aimed to explore the associations between TyG index with ischemic stroke recurrence in nondiabetic patients with small vessel occlusion.

**Methods:**

From November 1, 2016 to February 28, 2021, consecutive acute ischemic stroke patients admitted within 1 week after onset were screened. The stroke mechanism was determined based on medical history, laboratory examinations, cardiac examinations, vascular examinations and neuroimaging. Nondiabetic patients with small vessel occlusion were enrolled and followed up for 1 year. The primary outcome was ischemic stroke recurrence. Logistic regression and Kaplan–Meier survival curve were used to analyze the association of the TyG index and stroke recurrence.

**Results:**

A total of 6100 acute ischemic stroke patients were screened, with 1970 nondiabetic patients with small vessel occlusion included and divided into 4 groups according to the TyG index quartiles (Q1: < 8.20; Q2: 8.20–8.53; Q3: 8.54–8.92; Q4: > 8.92). There were significant differences in age, body mass index, systolic blood pression, diastolic blood pressure, lipid-lowering agents, infarct location, fasting blood glucose, total cholesterol, triglyceride, low-density lipoprotein cholesterol, high-density lipoprotein cholesterol, uric acid, and stroke recurrence among the 4 groups. In the multi-adjusted models, compared to Q1 of the TyG index, the odds ratio for Q4 of the TyG index for stroke recurrence was 3.100 (1.366–8.019). The Kaplan–Meier survival (ischemic stroke-free) curves by quartiles of the TyG index also showed statistically significant differences (log-rank test, *P* = 0.004).

**Conclusions:**

Our findings suggested that the TyG index was associated with ischemic stroke recurrence in nondiabetic patients with small vessel occlusion, and it could be a valuable biomarker for assessing the risk of ischemic stroke recurrence in these patients.

## Background

In the Trial of Org 10172 in Acute Ischemic Stroke Treatment (TOAST) classification system, small-vessel occlusion (SVO), also known as lacunar stroke, is attributed to cerebral small vessel disease (CSVD) affecting the deep penetrating arterioles [1]. Although with relatively mild symptoms on admission, SVO was reported to have similar long-term risks of all-cause mortality and stroke recurrence with non-SVO strokes [2]. Since recurrent ischemic stroke is usually more severe and disabling than the first episode, it is vital to identify the risk factors of stroke recurrence.

Type 2 diabetes mellitus has been recognized to be associated with a two- to six-fold increased risk for first or recurrent ischemic stroke [3, 4]. The underlying mechanisms are myriad and include insulin resistance which is universal in diabetic patients [5, 6]. A variety of literature demonstrates that insulin resistance augments the role of the modifiable risk factors of ischemic stroke and enhances platelet adhesion, activation and aggregation which are conducive to the occurrence and recurrence of ischemic stroke [7]. However, most of the SVO patients (63% in the Secondary Prevention of Small Subcortical Strokes (SPS3) study) do not have diabetes mellitus [3]. Insulin resistance is also prevalent in nondiabetic people [8]. Hence, it is necessary to verify the effect of insulin resistance on the ischemic stroke recurrence of nondiabetic patients with SVO.

Triglyceride-glucose (TyG) index, denoted ln [triglycerides (TG) (mmol/L) ^∗^ fasting blood glucose (FBG) (mmol/L)/2], is a simple and reliable surrogate marker of insulin resistance [9]. Many previous studies have shown an association between a higher TyG index and an increased incidence of atherosclerosis, intima media thickness of common carotid artery, unstable carotid plaque, hypertension, arterial stiffness, and cardiovascular disease [10–13]. As expected, a plenty of evidences proved that TyG index was independently associated with the occurrence of stroke, and a higher TyG index was also a predictor of all-cause mortality and poor outcomes in patients with acute ischemic stroke [14–17].

However, evidence on the correlation between the TyG index and stroke recurrence is scarce up to date. Moreover, results were inconsistent among studies. Zhou et al. observed that a higher TyG index was related to an increased risk of stroke recurrence in patients with ischemic stroke [18]. Yang et al. found that in a group of nondiabetic acute ischemic stroke patients, elevated TyG index was associated with an increased risk of stroke recurrence and death [19]. However, Nam et al. reported that TyG index was only associated with early recurrent ischemic lesions in acute ischemic stroke patients with large artery atherosclerosis, not in patients with other stroke mechanisms [20].

Therefore, the present study was conducted to investigate the relationship between TyG index and ischemic stroke recurrence in nondiabetic patients with SVO.

## Methods

### Study population

The research protocol of this hospital-based prospective cohort study was reviewed and approved by the ethics committees of each center (the Second Affiliated Hospital of Guangzhou Medical University, the First Affiliated Hospital of Soochow University, and Wuxi People’s Hospital) according to the principles expressed in the Declaration of Helsinki. Written consents were obtained from patients or their authorized representatives before enrollment.

### Experimental design

Patients with acute ischemic stroke admitted in the centers from November 1, 2016 to February 28, 2021 were consecutively screened. All patients underwent standard magnetic resonance imaging (MRI) scan including T1-weighted imaging, T2-weighted imaging, diffusion weighted imaging (DWI), fluid-attenuated inversion recovery sequence, and apparent diffusion coefficient maps within 7 days of stroke onset. Ultrasound and magnetic resonance angiography (MRA) or computed tomography angiography (CTA) or digital subtraction angiography (DSA) were applied to evaluate extracranial and intracranial arteries (stenosis, occlusion or dissection). Cardiac examinations (electrocardiogram and echocardiogram) were performed. Patients were enrolled if they met the inclusion criteria as follows: (1) older than 18 years; (2) acute ischemic stroke lesions confirmed by MRI; (3) with TOAST classification of SVO; (4) with the data of FBG and TG to calculate TyG index. Patients were excluded if they met any of the following criteria: (1) delay of > 7 days from symptom recognition to admission; (2) with diabetes mellitus; (3) with TOAST classification of large artery atherosclerosis, cardioembolism, other determined causes such as arterial dissection, or cryptogenic stroke (including ≥ 2 plausible causes); (4) missing clinical or imaging data, or poor quality of neuroimages; (5) with intracranial hemorrhage (including hemorrhage transformation); (6) the estimated remaining lifetime was less than 12 months.

### Data collection and follow up

Trained interviewers conducted face-to-face interviews at each center to collect data at baseline. On admission, demographic information (including age and gender), risk factors for stroke (including current smoking or drinking, body mass index (BMI), hypertension, systolic blood pressure (SBP), diastolic blood pressure (DBP), previous stroke or transient ischemic attack (TIA), and previous coronary artery disease), laboratory examinations [including FBG, total cholesterol, TG, high-density lipoprotein (HDL) cholesterol, low-density lipoprotein (LDL) cholesterol, and uric acid, performed after 12 h of overnight fasting] and medications on admission (including antihypertensive agents, lipid-lowering agents, antiplatelet agents, and anticoagulant agents) were acquired. Current drinking was defined as heavy intake (≥ 14 drinks/week in women or 21 drinks/week in men) or episodic heavy intake (≥ 5 drinks/episode at least once per month). Diabetes mellitus was defined as glycosylated hemoglobin ≥ 6.5%, or FBG levels ≥ 7.0 mmol/L, or blood glucose ≥ 11.1 mmol/L at 2 h by oral glucose tolerance test, or blood glucose ≥ 11.1 mmol/L at random glucose test, or any use of glucose-lowing drugs, or any self-reported history of diabetes. Hypertension was defined as systolic pressures ≥ 140 mmHg or diastolic pressure ≥ 90 mmHg, any use of antihypertensive drugs, or a self-reported history of hypertension. The TyG index was calculated using the formula of “the log scale of [TG (mmol/L) ^∗^ FBG (mmol/L)/2]”.

Enrolled patients received standard therapy including thrombolytic therapy, or other medications at the acute stage of stroke, and then standard secondary prevention including risk factors control, antiplatelet drugs and standard dose of statins. The start point of follow-up was the incident date of stroke onset. All patients were followed up for 12 months (at 1, 3, 6, and 12 months after stroke onset) by telephone or in the outpatient department. The primary outcome was ischemic stroke recurrence. If any suspected new neurological symptoms emerged, the patients would be evaluated by a senior neurologist. When a recurrence was suspected, MRI was performed within 1 week after symptoms emergence to verify the stroke lesion.

### Statistical analysis

Statistical analysis was performed using SPSS (version 20.0). Continuous variables were tested for normality using the Kolmogorov–Smirnov test. Normally distributed continuous variables were described as the mean ± standard deviation, while categorical variables were expressed as percentages. Group differences were analyzed using the Kruskal–Wallis test for continuous variables, and the chi-square test for categorical variables. The chi-square trend test was used to test the trends of the prevalence of ischemic stroke recurrence with increasing TyG index. Logistic regression was used to analyze the association of the TyG index and ischemic stroke recurrence by calculating odds ratio (OR) and 95% confidence interval (CI). Three models were constructed, Model 1 was unadjusted, Model 2 was adjusted for age, lipid-lowering agents, FBG, BMI, SBP, DBP, total cholesterol, TG, HDL cholesterol, LDL cholesterol, uric acid and infarct locations, Model 3 was further adjusted for gender, smoking, drinking, hypertension, antihypertensive agents, antiplatelet agents, anticoagulant agents, stroke or TIA history and coronary artery disease history. Kaplan–Meier event-free survival curves were generated and the significance was evaluated by log-rank tests. The *P* values for trend were computed using quartiles of TyG index as ordinal variable. A two-sided *P* < 0.05 was considered statistically significant.

## Results

### Baseline characteristics

**Fig. 1:**
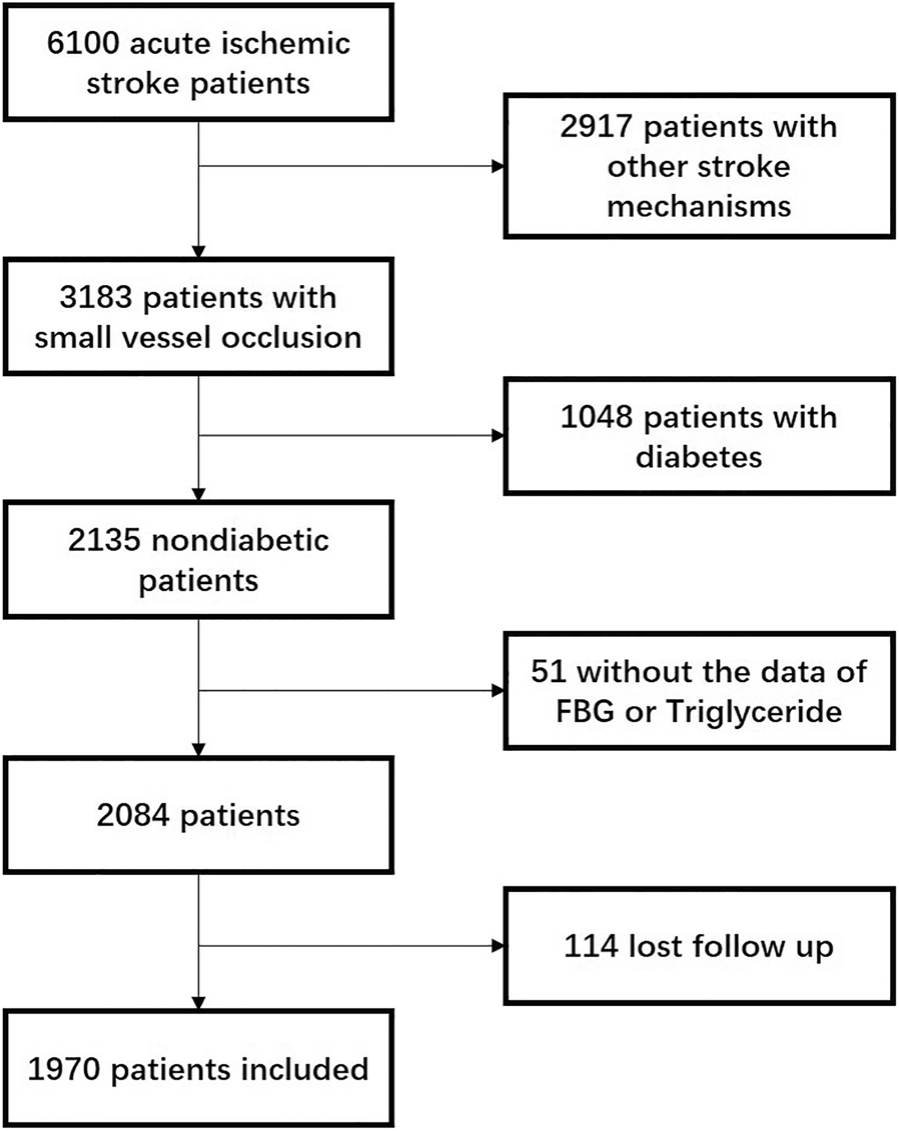
Flow chart of patient recruitment

In total, 6100 patients with acute ischemic stroke within 1 week of onset were consecutively screened. Among these patients, 2917 patients with other stroke mechanisms except for SVO were excluded. For the remaining 3183 patients with SVO, 1048 patients with diabetes mellitus were further excluded. Removing the data of patients without FBG or TG or patients missing during follow-up, a total of 1970 patients were included in the statistical analysis (Fig. 1).

The basic characteristics are presented in Table 1. The age of the participants was 66.46 ± 12.35 years old. Generally speaking, participants with a higher TyG index were more likely to be younger, to have lipid-lowering agents, to have a higher BMI, a higher SBP, a higher DBP, a higher prevalence of posterior circulation stroke, a higher level of FBG, total cholesterol, TG, LDL cholesterol, and uric acid but a lower HDL cholesterol level, compared with participants with a lower TyG index.

**Table 1:** Baseline characteristics of the study participants

Characteristics	Overall	Quartiles of the TyG index				*P*
		Q1 (< 8.20)	Q2 (8.20–8.53)	Q3 (8.54–8.92)	Q4 (> 8.92)	
No. of patients	1970	502	487	497	484	
Age (y)	66.46 ± 12.35	69.91 ± 12.95	67.38 ± 12.33	65.29 ± 11.97	63.17 ± 12.35	< 0.001
Male (%)	1345 (68.27%)	360 (71.71%)	339 (69.61%)	323 (64.99%)	323 (66.74%)	0.105
Smoking (%)	580 (29.44%)	136 (27.09%)	160 (32.85%)	142 (28.57%)	142 (29.33%)	0.236
Drinking (%)	217 (11.01%)	47 (9.36%)	60 (12.32%)	53 (10.66%)	57 (11.78%)	0.456
BMI (kg/m^2^)	26.75 ± 4.41	25.12 ± 4.17	26.29 ± 3.81	27.16 ± 5.07	28.40 ± 3.84	< 0.001
Hypertension (%)	1377 (69.90%)	332 (66.13%)	343 (70.43%)	350 (70.42%)	352 (72.73%)	0.148
SBP on admission (mmHg)	134.03 ± 26.16	124.17 ± 22.64	130.92 ± 22.96	137.33 ± 28.12	144.01 ± 26.34	< 0.001
DBP on admission (mmHg)	79.48 ± 11.37	73.33 ± 8.55	77.07 ± 9.97	79.35 ± 9.79	88.43 ± 11.22	< 0.001
Antihypertensive agents (%)	1122 (56.95%)	281 (55.98%)	280 (57.49%)	282 (56.74%)	279 (57.64%)	0.965
Lipid-lowering agents (%)	1642 (83.35%)	393 (78.29%)	400 (82.13%)	427 (85.92%)	422 (87.19%)	0.001
Antiplatelet agents (%)						0.110
DAPT	1257 (63.81%)	322 (64.14%)	328 (67.35%)	299 (60.16%)	308 (63.64%)	
MAPT	471 (23.91%)	114 (22.71%)	99 (20.33%)	131 (26.39%)	127 (26.24%)	
Anticoagulant agents (%)	53 (2.69%)	14 (2.79%)	17 (3.49%)	10 (2.01%)	13 (2.68%)	0.567
Fasting blood glucose (mM)	5.08 ± 1.33	4.53 ± 0.86	4.80 ± 0.73	5.15 ± 0.93	5.85 ± 2.01	< 0.001
Total cholesterol (mM)	4.63 ± 1.10	4.12 ± 0.96	4.49 ± 0.98	4.86 ± 1.09	5.08 ± 1.14	< 0.001
Triglyceride (mM)	1.57 ± 1.07	0.80 ± 0.18	1.16 ± 0.19	1.54 ± 0.28	2.81 ± 1.48	< 0.001
HDL cholesterol (mM)	1.08 ± 0.28	1.18 ± 0.32	1.09 ± 0.25	1.06 ± 0.26	0.98 ± 0.26	< 0.001
LDL cholesterol (mM)	3.05 ± 0.98	2.64 ± 0.84	3.00 ± 0.89	3.32 ± 1.00	3.27 ± 1.01	< 0.001
Uric acid (μM)	360.72 ± 104.39	337.30 ± 100.06	351.79 ± 102.91	362.50 ± 101.41	392.08 ± 105.76	< 0.001
Stroke or TIA history (%)	366 (18.58%)	108 (21.51%)	97 (19.92%)	85 (17.10%)	76 (15.70%)	0.079
CHD history (%)	153 (7.77%)	43 (8.56%)	43 (8.83%)	33 (6.64%)	34 (7.02%)	0.474
Infarct location (%)						< 0.001
Anterior circulation	1322 (67.11%)	367 (73.11%)	331 (67.97%)	332 (66.80%)	292 (60.33%)	
Posterior circulation	648 (32.89%)	135 (26.89%)	156 (32.03%)	165 (33.20%)	192 (39.67%)	
Ischemic stroke recurrence (%)	115 (5.84%)	14 (2.79%)	25 (5.13%)	34 (6.84%)	42 (8.68%)	0.001
CE	11 (9.56%)	2 (14.28%)	3 (12.00%)	3 (8.82%)	3 (7.14%)	0.455
LAA	25 (21.74%)	3 (21.42%)	2 (8.00%)	11 (32.35%)	9 (21.43%)	
SVO	79 (68.70%)	9 (64.28%)	20 (80.00%)	20 (58.82%)	30 (71.43%)	

*TyG* triglyceride-glucose, *BMI* body mass index, *SBP* systolic blood pression, *DBP* diastolic blood pressure, *DAPT* dual antiplatelet therapy, *MAPT* mono antiplatelet therapy, *HDL* high-density lipoprotein cholesterol, *LDL* low-density lipoprotein cholesterol, *TIA* transient ischemic attack, *CHD* coronary artery disease, *CE* cardio-embolism, *LAA* large-artery atherosclerosis, *SVD* small-vessel occlusion

### Association of the TyG index with stroke recurrence

**Fig. 2:**
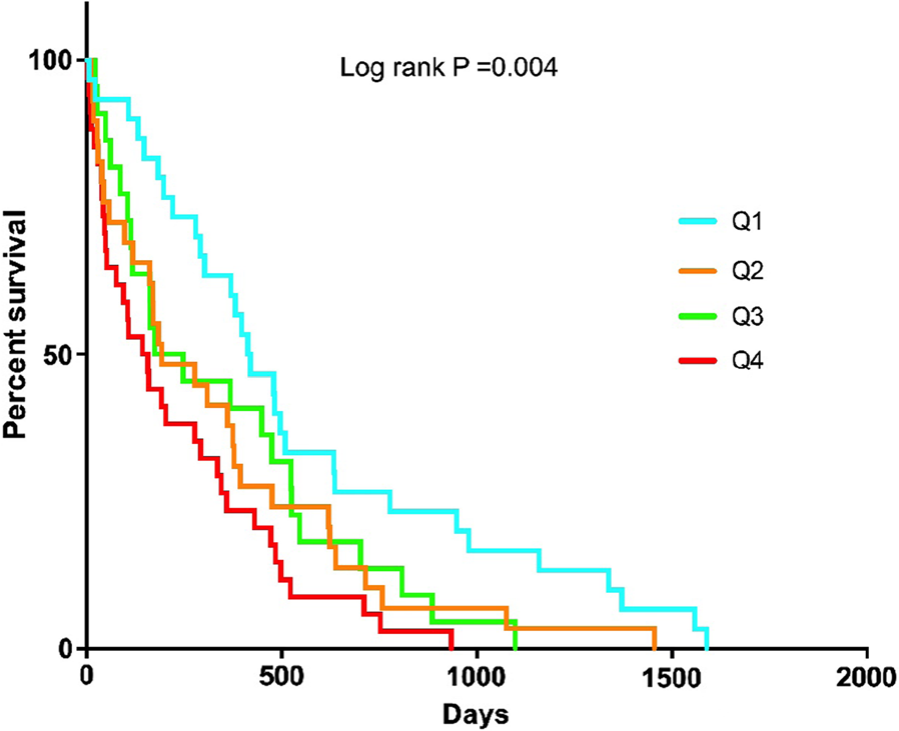
The Kaplan–Meier event-free survival analysis according to the quartiles of TyG index. TyG index, triglyceride-glucose index

As shown in Table 1, ischemic stroke recurrence occurred in 115 (5.84%) patients in total. With increasing TyG index quartiles, the prevalence of recurrent ischemic stroke increased substantially, reaching to a prevalence of 8.68% in the highest quartile of TyG index (*P* for trend < 0.001). In the multivariate logistic regression analysis, when the TyG index was used as a continuous variable, it was significantly associated with the risk of ischemic stroke recurrence, per 1 unit increase of the TyG index was associated with 4.738-fold higher prevalence of stroke recurrence (*P* = 0.003, OR 4.738, 95% CI 1.722–13.306. The positive association between the TyG index and ischemic stroke recurrence remained significant when the TyG index was used as a categorical variable in multivariate logistic regression analysis. The adjusted OR was 2.596 (95% CI 1.284–5.248) for the Q2 quartile, 3.836 (95% CI 1.850–7.952) for the Q2 quartile, and 3.100 (95% CI 1.366–8.019) for the highest quartile of TyG index, compared to the lowest quartile of TyG index (Table 2). The Kaplan–Meier event-free survival analysis indicated that the cumulative incidence of ischemic stroke recurrence increased incrementally across quartiles of the TyG index (log-rank test, *P* = 0.004, Fig. 2), and patients within the highest quartile of TyG index presented the lowest event-free survival.

**Table 2:** Multivariate adjusted ORs (95% CI) of the association of TyG index with ischemic stroke recurrence

Model	Quartiles of the TyG index				*P*
	Q1 (< 8.20)	Q2 (8.20–8.53)	Q3 (8.53–8.92)	Q4 (> 8.92)	
Model 1	Reference	1.886 (0.969–3.673)	2.560 (1.356–4.831)	3.312 (1.785–6.147)	0.001
Model 2	Reference	2.418 (1.205–4.855)	3.523 (1.721–7.211)	2.975 (1.255–7.051)	0.008
Model 3	Reference	2.596 (1.284–5.248)	3.836 (1.850–7.952)	3.100 (1.366–8.019)	0.004

Model 1: unadjusted. Model 2: adjusted for age, lipid-lowering agents, fasting blood glucose, body mass index, systolic blood pressure, diastolic blood pressure, total cholesterol, triglyceride, high-density lipoprotein cholesterol, low-density lipoprotein cholesterol, uric acid and infarct locations. Model 3: further adjusted for gender, smoking, drinking, hypertension, antihypertensive agents, antiplatelet agents, anticoagulant agents, stroke or transient ischemic attack history and coronary artery disease history

*TyG* triglyceride-glucose, *OR* odds ratio, *CI* confidence interval

## Discussion

In this multicenter hospital-based study, we found that 1 year recurrence rate of nondiabetic patients with SVO was 5.84%, and the TyG index was independently associated with ischemic stroke recurrence in these patients. Our results revealed that the TyG index was a valuable biomarker for assessing the risk of ischemic stroke recurrence in nondiabetic patients with SVO, and more attention should be paid to these patients with higher TyG indices for the secondary prevention of stroke.

Several previous studies indirectly supported our result of the relationship between TyG index and stroke recurrence. Zhou et al. concluded that TyG index was associated with an increased risk of stroke recurrence, all-cause mortality, and neurologic worsening in diabetic and nondiabetic patients with ischemic stroke [18]. Yang et al. reported that elevated TyG index was associated with an increased risk of stroke recurrence and death in nondiabetic stroke patients, regardless of stroke mechanism [19]. Recently, Wang et al. found that elevated TyG index was associated with stroke recurrence in elderly patients (more than 65 years) with ischemic stroke [21]. However, Nam et al. found a close correlation between TyG index and early recurrent ischemic lesions in patients with the large artery atherosclerosis, but not in patients with other stroke mechanisms [20]. This conclusion was inconsistent with our result. The possible reason might be that Nam et al. only included 176 acute ischemic stroke patients in total.

The correlation between a higher TyG index and an elevated risk of ischemic stroke recurrence could be attributed to insulin resistance, a prevalent and important risk factor of ischemic stroke [22]. The gold standard marker of insulin resistance, homeostasis model assessment-insulin resistance (HOMA-IR) index was independently associated with poor functional outcome after acute ischemic stroke, and with an increased risk of death, stroke recurrence and poor outcome in nondiabetic patients with acute ischemic stroke [7, 23–25]. The Insulin Resistance Intervention after Stroke (IRIS) tested the hypothesis that pioglitazone, a glucose-lowering insulin-sensitizing drug used for the treatment of insulin resistance (a score of more than 3.0 on the HOMA-IR index), would reduce the rates of stroke and myocardial infarction after ischemic stroke or TIA in nondiabetic patients with insulin resistance, and achieved positive findings [5]. Although widely used in the research field, the application of HOMA-IR (calculated as fasting insulin (μU/mL) × fasting glucose (mmol/L)/22.5) is highly limited in clinical practice because of the need for insulin measurement, which is expensive and not available in many hospitals, especially in undeveloped countries [26]. In 2008, Simental-Mendía et al. developed the TyG index to estimate insulin resistance, based on the concept that insulin resistance also includes impairment in the oxidation and utilization of fatty acids, and verified the TyG index was a reliable and reproducible index to measure insulin resistance in healthy subjects [27]. Since then, TyG index has been recognized as a reliable surrogate biomarker of insulin resistance and has been proposed and validated to be highly correlated with HOMA-IR across all the ages and various ethnic groups [28–30].

Except for the direct effect of insulin resistance, there might be some other explanations for the association of TyG index and stroke recurrence in nondiabetic patients with SVO. First, TyG index has been related to hypertension, and hypertension was reported to double the risk of recurrent stroke in patients with lacunar stroke [31]. Second, insulin resistance is a key pathological process during the development of CSVD [24, 32]. Predictably, TyG index has been proved to be positively associated with a higher prevalence and burden of CSVD [9, 33–36], which was reported to predict a recurrent ischemic stroke or intracerebral hemorrhage in numerous studies [1, 37–41]. CSVD could make a contribution to the mechanism of increasing ischemic stroke recurrence by a higher TyG index. Third, insulin resistance is an independent predictor for development of antiplatelet drug resistance which leads to the failure of secondary stroke prevention in a large proportion of stroke patients [7, 23]. Finally, TyG index is related to other vascular risk factors such as unstable carotid plaques which might also lead to recurrent ischemic stroke [10–13].

Our study has several limitations. First, first and recurrent ischemic strokes were strictly defined according to the MRI findings. This would cause potential underestimate of stroke occurrence and recurrence, since there might be a small proportion of DWI-negative strokes and TIA. However, we performed MRI on all patients with suspected stroke to determine stroke mechanism and stroke recurrence. Because, some stroke symptoms like isolated vertigo are difficult to be distinguished from other benign diseases. MRI is indispensable under this circumstance. Moreover, infarcts sometimes occur at the same or adjacent sites with the first strokes, especially when the recurrent stroke mechanism is the same with the first one [42]. In this case, the recurrent infarcts need to be verified by DWI. Second, the annual risk of developing diabetes type 2 in people with normal glucose level is 0.7%, whereas patients with prediabetes have a yearly risk of up to 10% [43]. During the 1 year follow-up, there should be a few patients newly diagnosed as diabetes, which might cause some bias to the relationship between the TyG index and stroke recurrence in nondiabetic patients, however, we did not exclude this part of patients in the present study. Lastly, the determination of SVO as the stroke mechanism has always been ambiguous ever since the TOAST classification system was established, there were probably some patients with other mechanisms misdiagnosed as SVO, and vice versa.

## Conclusions

In summary, our study found that the TyG index was positively and independently associated with recurrent ischemic stroke in nondiabetic patients with SVO. This finding indicates that the TyG index could be a useful biomarker in early recognition of patients at a high risk of ischemic stroke recurrence, and more attention should be paid in the secondary stroke prevention in these patients.

## Data Availability

The datasets used and/or analyzed during the current study are available from the corresponding author on reasonable request.

## Abbreviations

TOAST: Trial of org 10172 in acute ischemic stroke treatment
SVO: Small-vessel occlusion
CSVD: Cerebral small vessel disease
SPS3: Secondary prevention of small subcortical strokes
TyG: Triglyceride-glucose
TG: Triglycerides
FBG: Fasting blood glucose
MRI: Magnetic resonance imaging
DWI: Diffusion weighted imaging
MRA: Magnetic resonance angiography
CTA: Computed tomography angiography
DSA: Digital subtraction angiography
BMI: Body mass index
SBP: Systolic blood pressure
DBP: Diastolic blood pressure
HDL: High-density lipoprotein
LDL: Low-density lipoprotein
TIA: Transient ischemic attack
OR: Odds ratio
CI: Confidence interval
HOMA-IR: Homeostasis model assessment-insulin resistance
IRIS: Insulin resistance intervention after stroke

## References

[1] Mac Grory B, Yaghi S, Cordonnier C, Sposato LA, Romano JG, Chaturvedi S (2022). Advances in recurrent stroke prevention: focus on antithrombotic therapies. Circ Res.

[2] Portegijs S, Ong AY, Halbesma N, Hutchison A, Sudlow CL, Jackson CA (2022). Long-term mortality and recurrent vascular events in lacunar versus non-lacunar ischaemic stroke: a cohort study. Eur Stroke J.

[3] Palacio S, McClure LA, Benavente OR, Bazan C, Pergola P, Hart RG (2014). Lacunar strokes in patients with diabetes mellitus: risk factors, infarct location, and prognosis: the secondary prevention of small subcortical strokes study. Stroke.

[4] Kernan WN, Inzucchi SE (2004). Type 2 diabetes mellitus and insulin resistance: stroke prevention and management. Curr Treat Options Neurol.

[5] Kernan WN, Viscoli CM, Furie KL, Young LH, Inzucchi SE, Gorman M (2016). Pioglitazone after ischemic stroke or transient ischemic attack. N Engl J Med.

[6] Hadwen J, Kim W, Dewar B, Ramsay T, Davis A, Dowlatshahi D (2021). Association between insulin resistance and post-ischaemic stroke outcome in patients without diabetes: protocol for a systematic review and meta-analysis. BMJ Open.

[7] Deng XL, Liu Z, Wang C, Li Y, Cai Z (2017). Insulin resistance in ischemic stroke. Metab Brain Dis.

[8] Kernan WN, Inzucchi SE, Viscoli CM, Brass LM, Bravata DM, Shulman GI (2003). Impaired insulin sensitivity among nondiabetic patients with a recent TIA or ischemic stroke. Neurology.

[9] Teng Z, Feng J, Dong Y, Xu J, Jiang X, Chen H (2022). Triglyceride glucose index is associated with cerebral small vessel disease burden and cognitive impairment in elderly patients with type 2 diabetes mellitus. Front Endocrinol.

[10] Wang A, Li Y, Zhou L, Liu K, Li S, Song B (2022). Triglyceride-glucose index is related to carotid plaque and its stability in nondiabetic adults: a cross-sectional study. Front Neurol.

[11] Miao M, Zhou G, Bao A, Sun Y, Du H, Song L (2022). Triglyceride-glucose index and common carotid artery intima-media thickness in patients with ischemic stroke. Cardiovasc Diabetol.

[12] Liu Q, Cui H, Ma Y, Han X, Cao Z, Wu Y (2022). Triglyceride-glucose index associated with the risk of cardiovascular disease: the Kailuan study. Endocrine.

[13] Won KB, Park GM, Lee SE, Cho IJ, Kim HC, Lee BK (2018). Relationship of insulin resistance estimated by triglyceride glucose index to arterial stiffness. Lipids Health Dis.

[14] Hu L, Bao H, Huang X, Zhou W, Wang T, Zhu L (2022). Relationship between the triglyceride glucose index and the risk of first stroke in elderly hypertensive patients. Int J Gen Med.

[15] Zhao Y, Zhang J, Chen C, Qin P, Zhang M, Shi X (2022). Comparison of six surrogate insulin resistance indexes for predicting the risk of incident stroke: the rural chinese cohort study. Diabetes Metab Res Rev..

[16] Wang X, Feng B, Huang Z, Cai Z, Yu X, Chen Z (2022). Relationship of cumulative exposure to the triglyceride-glucose index with ischemic stroke: a 9 year prospective study in the Kailuan cohort. Cardiovasc Diabetol.

[17] Ma X, Han Y, Jiang L, Li M (2022). Triglyceride-glucose index and the prognosis of patients with acute ischemic stroke: a meta-analysis. Horm Metab Res.

[18] Zhou Y, Pan Y, Yan H, Wang Y, Li Z, Zhao X (2020). Triglyceride glucose index and prognosis of patients with ischemic stroke. Front Neurol.

[19] Yang X, Wang G, Jing J, Wang A, Zhang X, Jia Q (2022). Association of triglyceride-glucose index and stroke recurrence among nondiabetic patients with acute ischemic stroke. BMC Neurol.

[20] Nam KW, Kwon HM, Lee YS (2021). High triglyceride-glucose index is associated with early recurrent ischemic lesion in acute ischemic stroke. Sci Rep.

[21] Wang F, Wang J, Han Y, Shi X, Xu X, Hou C (2022). Triglyceride-glucose index and stroke recurrence in elderly patients with ischemic stroke. Front Endocrinol.

[22] Chen W, Wang S, Lv W, Pan Y (2020). Causal associations of insulin resistance with coronary artery disease and ischemic stroke: a mendelian randomization analysis. BMJ Open Diabetes Res Care.

[23] Jia W, Jia Q, Zhang Y, Zhao X, Wang Y (2021). Association between insulin resistance and aspirin or clopidogrel resistance in Chinese patients with recent ischemic stroke/TIA. Neurol Res.

[24] Jing J, Pan Y, Zhao X, Zheng H, Jia Q, Mi D (2017). Insulin resistance and prognosis of nondiabetic patients with ischemic stroke: the ACROSS-China study (abnormal glucose regulation in patients with acute stroke across China). Stroke.

[25] Ago T, Matsuo R, Hata J, Wakisaka Y, Kuroda J, Kitazono T (2018). Insulin resistance and clinical outcomes after acute ischemic stroke. Neurology.

[26] Lin SF, Hu HH, Chao HL, Ho BL, Chen CH, Chan L (2022). Triglyceride-glucose index and intravenous thrombolysis outcomes for acute ischemic stroke: a multicenter prospective-cohort study. Front Neurol.

[27] Simental-Mendia LE, Rodriguez-Moran M, Guerrero-Romero F (2008). The product of fasting glucose and triglycerides as surrogate for identifying insulin resistance in apparently healthy subjects. Metab Syndr Relat Disord.

[28] Du T, Yuan G, Zhang M, Zhou X, Sun X, Yu X (2014). Clinical usefulness of lipid ratios, visceral adiposity indicators, and the triglycerides and glucose index as risk markers of insulin resistance. Cardiovasc Diabetol.

[29] Lim J, Kim J, Koo SH, Kwon GC (2019). Comparison of triglyceride glucose index, and related parameters to predict insulin resistance in Korean adults: an analysis of the 2007–2010 Korean national health and nutrition examination survey. PLoS ONE.

[30] Locateli JC, Lopes WA, Simoes CF, de Oliveira GH, Oltramari K, Bim RH (2019). Triglyceride/glucose index is a reliable alternative marker for insulin resistance in South American overweight and obese children and adolescents. J Pediatr Endocrinol Metab.

[31] Arboix A, Blanco-Rojas L, Marti-Vilalta JL (2014). Advancements in understanding the mechanisms of symptomatic lacunar ischemic stroke: translation of knowledge to prevention strategies. Expert Rev Neurother.

[32] Jiang T, Zhou Y, Zhang D, Cao Z, Bian L, Ni Y (2021). Association of serum interleukin-34 and insulin resistance with cognitive impairment in patients with cerebral small vessel disease. Curr Neurovasc Res.

[33] Nam KW, Kwon HM, Jeong HY, Park JH, Kwon H, Jeong SM (2020). High triglyceride-glucose index is associated with subclinical cerebral small vessel disease in a healthy population: a cross-sectional study. Cardiovasc Diabetol.

[34] Jung DH, Park B, Lee YJ (2022). Relationship of the triglyceride-glucose index with subclinical white matter hypersensitivities of presumed vascular origin among community-dwelling Koreans. Int J Gen Med.

[35] Cai Y, Chen B, Zeng X, Xie M, Wei X, Cai J (2022). The triglyceride glucose index is a risk factor for enlarged perivascular space. Front Neurol.

[36] Zhang J, Hu M, Jia Y, Zhao S, Lv P, Fan M (2022). The triglyceride glucose index is associated with the cerebral small vessel disease in a memory clinic population. J Clin Neurosci.

[37] Tian Y, Pan Y, Yan H, Meng X, Zhao X, Liu L (2022). Coexistent cerebral small vessel disease and multiple infarctions predict recurrent stroke. Neurol Sci.

[38] Pantoni L (2010). Cerebral small vessel disease: from pathogenesis and clinical characteristics to therapeutic challenges. Lancet Neurol.

[39] Hao Z, Chen Y, Wright N, Qin H, Turnbull I, Guo Y (2021). Natural history of silent lacunar infarction: 10 year follow-up of a community-based prospective study of 0.5 million Chinese adults. Lancet Reg Health West Pac.

[40] Umeno T, Yamashita A, Mizota T, Uramatsu T, Matsuo T (2022). Predictive value of total small-vessel disease score for recurrent stroke in patients undergoing maintenance hemodialysis. J Stroke Cerebrovasc Dis.

[41] Lau KK, Li L, Schulz U, Simoni M, Chan KH, Ho SL (2017). Total small vessel disease score and risk of recurrent stroke: validation in 2 large cohorts. Neurology.

[42] Wu L, Li Y, Ye Z, Liu D, Dai Z, Zhu J (2022). Site and mechanism of recurrent pontine infarction: a hospital-based follow-up study. Brain Sci.

[43] Roquer J, Rodriguez-Campello A, Cuadrado-Godia E, Giralt-Steinhauer E, Jimenez-Conde J, Degano IR (2014). Ischemic stroke in prediabetic patients. J Neurol.

